# The Promise of Anti-idiotype Revisited

**DOI:** 10.3389/fimmu.2019.00808

**Published:** 2019-04-12

**Authors:** Heinz Kohler, Anastas Pashov, Thomas Kieber-Emmons

**Affiliations:** ^1^Department of Microbiology and Immunology, University of Kentucky, Lexington, KY, United States; ^2^Stephan Angelov Institute of Microbiology, Bulgarian Academy of Sciences, Sofia, Bulgaria; ^3^Department of Pathology, Winthrop P. Rockefeller Cancer Institute, University of Arkansas for Medical Sciences, Little Rock, AR, United States

**Keywords:** idiotype, polyclonal, vaccines, polyreactive, multi-epitope binding, therapeutic, mimetic

## Abstract

The promise of idiotype-based therapeutics has been disappointing forcing a new look at the concept and its potential to generate an effective approach for immunotherapy. Here, the idiotype network theory is revisited with regard to the development of efficacious anti-idiotype vaccines. The experience of polyclonal anti-Idiotype reagents in animal models as well as an understanding of the immune response in humans lends to the proposition that polyclonal anti-Idiotype vaccines will be more effective compared to monoclonal-based anti-Idiotype vaccines. This novel strategy can be adapted in Biotech-standard production of therapeutic antibodies.

## 1. Introduction

The strategy of using anti-idiotype (anti-Id) antibodies as surrogate antigens stems from the Idiotype cascade proposed by Niels Jerne (1). Accordingly, anti-Id antibodies were originally described as Ab2α and Ab2β, whereby the former does not block antigen binding and the latter can inhibit binding of the corresponding Ab1 to its antigen. This lent to the conclusion that Ab2β mimics structurally the antigen for Ab1. The concept and its experimental use have been extensively reviewed, (2–5). Noted advantages of using anti-Ids over nominal antigens as therapeutic vaccines include difficulties to produce vaccines containing non-protein antigens. Anti-Ids can be produced that mimic lipid, carbohydrate or nucleic acid epitopes or even drugs. Tolerance to antigens is a major hurdle in vaccine development. Antibody-B Cell Receptor binding occurs at multiple sites, while antigen strictly binds to Complementary Determining Regions (CDRs) of antibodies. This allows stimulation of a broader determinate targeting antibody response that might include epitope spreading. Finally, anti-Ids can be persistent in inducing an immune response against antigens while avoiding autoimmune responses triggered by nominal antigen based vaccines (6).

A major obstacle both theoretically and practically is reconciling the immunization concept with the postulated restriction of the putative idiotypic network of natural antibody producing B cell clones (7). Natural antibodies, in the strictest sense, are constitutively produced (8), but this strict definition leaves out some polyreactive antibodies induced in marginal zone B cells and in T-cell independent responses, which can also be defined as natural antibodies in a broader sense (9–11). The gray zone of the natural antibody concept probably contains the answers to some of the paradoxes of idiotypy. Thus, several animal studies using anti-Id antibodies support their utility, as vaccines while human trials with monoclonal Ab2β were disappointing and have failed in later phase trials. Here, we analyze this failure and propose an alternative strategy for an idiotype-based immunotherapy.

## 2. Setting the Stage for the Idiotype Interactions in Regulating an Immune Response

In 1963 two laboratories reported evidence for a new marker on antibodies distinct from allotypes (12, 13). The term IDIOTYPE for determinants recognized by antibodies was adopted. Recognizing that antibodies against antibodies exist and playing a number game on the multitude of B-cells producing antibodies, Jerne concluded that there must be a functional network of idiotype (Id) and anti-idiotypes (anti-Id) (14). Thus, the idiotype network hypothesis was born. Yet, evidence was lacking for network interactions during an induced immune response and that an anti-Id response might have a regulating function. In 1972 several reports appeared on the potential of anti-Id antibodies to suppress a specific immune response (15–17). Such results suggested that anti-Ids can affect an immune response, but did not establish that immune-modulation is part of an antigen-induced immune response. Two reports supported this latter premise (17, 18). An idiotypic cascade was perceived: Ab1>Ab2β >Ab3. Ab3 would resemble Ab1 and were labeled Ab1'. Jerne distinguished two types of anti-Ids (1, 14): Based on this concept, Ab2β's resemble structurally the antigen; thus the term *Internal Image* of antigen emerged as an explanation for this mimicry.

Shortly after this concept emerged several laboratories put this to the test by using Ab2β as antigen to induce target-specific immune responses (19–23). The dual functional property of Ab2 was demonstrated as either suppression (15) or induction of a specific response (24) to be dependent on the IgG-class (25). The idiotypic cascade implies that Ab1 used therapeutically might induce an antigen specific antibody response (26). Clinically, support for the idiotypic cascade is suggested in that patients developing low-level Human Anti-Mouse Antibody (HAMA) to a GD2 reactive Ab1 were shown to have higher long-term survival rates than those who did not (27, 28). GD2 is a disialoganglioside expressed on tumors of neuroectodermal origin, including human neuroblastoma and melanoma, with highly restricted expression on normal tissues, principally to the cerebellum and peripheral nerves in humans. The relatively tumor specific expression of GD2 makes it a suitable target for monoclonal antibody therapy and potentially a proving ground to probe and dissect network interactions.

The idiotype cascade has been suggested to be part of the functional utility of at least one monoclonal antibody presently approved by the US FDA [dinutuximab targeting the GD2 antigen: (29)]. The FDA approved Dinutuximab (Ch14.18, trade name Unituxin) and Dinutuximab beta (trade name Isquette), a monoclonal antibody used as a second-line treatment for children with high-risk neuroblastoma. However, differences in immune responses to Ab1 might be attributed to differences in Germline origins of the selected monoclonal Ab1 used in therapeutic application. A clinical trial with Ch14.18, a chimeric, in combination with IL-2, while showing a strong activation of antibody effector functions, did not show a better clinical outcome (30). Development of human anti-chimeric antibody (HACA) (21% of patients) did result in strong reduction of ch14.18 levels, abrogating complement dependent cytotoxicity and antibody dependent cellular cytotoxicity (31). The monoclonal studied in Cheung et al. (27, 28) is of the IGVH2-9^*^02 germline while the ch14.18 variable region is derived from the IGHV1S135^*^01 germ line. Little attention is paid to such difference yet we know that no two antibodies need to be alike immunologically.

## 3. Lessons Learned from Therapeutic Anti-Id Antibodies

While the earlier anti-Id data were generated with polyclonal antibodies, later experiments used monoclonal anti-Ids (32, 33). The successful use of monoclonal anti-Ids as vaccines in inbred mice prompted several clinical trials with monoclonal Ab2β antibodies. The early studies on the immunomodulatory activities of Ab2, while consistently demonstrating immunological activity in animals, clinical trials with anti-Ids in the cancer space proved to be mixed (34). Herlyn and coworkers demonstrated that humoral immune reactivity against a tumor can be enhanced upon active anti-id vaccination (35). In these studies 30 patients with advanced colorectal carcinoma (CRC) were treated with alum-precipitated polyclonal goat anti-Id antibodies to monoclonal anti-CRC antibody CO17-1A (Ab1) in doses between 0.5 and 4 mg per injection. All patients developed Ab3 with binding specificities on the surface of cultured tumor cells similar to the specificity of Ab1. Furthermore, the Ab3 competed with Ab1 for binding to CRC cells. Fractions of Ab3-containing sera obtained after elution of the serum immunoglobulin from CRC cells bound to purified tumor antigen and inhibited binding of Ab2 to Ab1. Six patients showed partial clinical remission and seven patients showed arrest of metastases following immunotherapy (35). Therefore, it was concluded that the Ab3 could share binding similarities with Ab1.

In other studies, an anti-Id vaccine to induce anti-Carcinoembryonic antigen (CEA) antibodies (Ab3) was tested in non-human primates (36). CEA is a tumor marker largely utilized for the detection of minimal disease associated with colon cancer and considered a target for immunotherapy. The murine monoclonal antibody specific for CEA, was generated via hybridoma technology and selected for inhibition of the binding to CEA. These successful preclinical studies led to clinical trials in humans with CEA positive tumors (37). In this trial, 9 of 12 patients demonstrated an anti-anti-idiotypic (Ab3) response. All nine patients generated specific anti-CEA antibody demonstrated by reactivity with radiolabeled purified CEA. Toxicity was limited to local reaction with mild fever and chills. However, in all 12 patients the tumor progressed after completion of the trial. Four of seven responding patients were reported to have T cell responses to purified CEA suggesting that there was an antigen specific T cell response after immunization (37). A patent was filed for the anti-Id (Chatterjee et al. 5,977,315). Yet, a phase II trial with anti-Id did not improve relapse of tumor (38) and a phase III study with the anti-Id and 5-Fluorouracil (5-FU) did not improve the overall outcome of the study (39). In preclinical models CEA was found to be up-regulated after exposure of cancer cells to 5-FU (40). Therefore, the premise for combination therapy would be to increase the expression of the target antigen for Ab3 to bind to.

Further anti-Id-based vaccine studies in humans have included those associated with Tumor Associated Carbohydrate Antigens (TACAs), particularly the ganglioside targets GD3 and GD2. The anti-Id BEC2, a mimic for GD3, was found not to be highly immunogenic in melanoma patients suggesting adjuvants might be necessary (41–43). More recently BEC2 was considered as a therapeutic intervention in GBS by neutralizing specific pathogenic anti-ganglioside antibodies (44). The murine monoclonal anti-Id antibody 1A7 (TriGem), a mimic of GD2, has been tested in pre-clinical studies and in the clinic (45). In pre-clinical studies, active immunization of mice, rabbits, and monkeys with TriGem induced polyclonal IgG anti-GD2 responses and TriGem specific T cell proliferative responses suggesting the generation of CD4+ T cell help. In clinical trials, it was demonstrated that patients with advanced metastatic melanoma and patients with high-risk melanoma in the postsurgical adjuvant setting generated active immune responses against GD2 following immunization with TriGem. IgG subclasses were shown to be predominately IgG1 and IgG4, suggesting the possibility of the generation of CD4+ T cell help. Median survival was 16+ months for 47 patients with advanced disease. Eighty-two percent of 69 patients with stage III disease were alive at a median follow up of 2 years.

An anti-Id vaccine has reached the market. Racotumomab (Vaxira) is now the first approved anti-Id vaccine—with approval in Cuba and Argentina. Vaxira was shown to increase the survival of Non-Small Cell Lung Cancer patients in recurrent or advanced stages (IIIB/IV). A phase III trial is currently ongoing (NCT01460472). The vaccine was initiated by the Center for Molecular Immunology in Havana, Cuba. Racotumomab, an Ab2γ, was raised against the murine anti-ganglioside N-glycolyl (NGc) GM3 (NGcGM3) (46). The safety of Racotumomab was established in several phase I trials in melanoma, breast and lung cancers (47, 48). In the lung trial, patients developed antibodies against NGcGM3 and had longer medium survival times (49). Results from a randomized trial with Racotumomab showed necrosis of tumor cells as a mechanism for efficacy (50).

While preclinical studies suggested that anti-Ids could mediate cellular responses, little evidence in humans demonstrates this aspect (51, 52). The most direct example for the activation of CD8+ Cytotoxic T Lymphocytes (CTL) involvement comes from a clinical trial testing a combination of the murine anti-id monoclonal antibodies MEL-2 and MF11–30 that are mimics of the high molecular weight melanoma-associated antigen (HMW-MAA) (53). The two anti-ids mimic two distinct epitopes of HMW-MAA. This combination called MELIMMUNE was shown to induce HLA-A2-restricted CTLs that lyse melanoma cells expressing both HLA-A2 antigen and HMW-MAA (53). Collectively, preclinical and clinical trials, albeit very limited, indicate that anti-Id vaccines can induce B and T-cell immune responses both in general terms supporting CD4+ T cell activation for IgG production and tumor antigen specific CD8+ CTLs if the anti-Ids are properly chosen.

## 4. Solving the Problems With Current Anti-Id Vaccines

While showing promise, to date no anti-Id-based vaccines has been approved by the US FDA for use in patients. Reasons for the failure of anti-Id vaccines against tumors are similar to generalized failures of other cancer vaccines. On the one hand it is possible that such failures reflect the patient populations used in the studies. We have now come to realize that checkpoint inhibitors are necessary to take the brakes off the immune system. On the other hand a major problem in cancer is the complexity and heterogeneity of antigen expression, the antigens that are potential targets of T and B-cells are multiple, diverse and endlessly adaptable. This reduces the ability of responding immune cells to consistently carry out their task to recognize, bind and destroy. A lesson might be forthcoming from consideration of the “normal” immune response to pathogens as many viruses, bacteria, and parasites induce a strong polyclonal B cell response, which can be crucial for early host defense against rapidly dividing microorganisms. In certain situations the response is restricted such as in HIV infections (54, 55). Interestingly, this clonal-restricted antibody response shares an idiotypic marker (56), termed Ab2δ. The polyclonal and sometimes oligoclonal antibodies in immune reactions would suggest that, in order to stimulate the polyclonal Ab1 spectrum, Ab2 should also be polyclonal. Early vaccine experiments were performed in rabbits and not subject to potential monoclonal anti-Id restrictions (25, 57). Later experiments suggested a strategy to simulate polyclonal immunization by combining monoclonals that are functional anti-Ids in that they compete with antigen clearly are not distinguished in their ability to activate functional T cell responses *a priori* (53, 58, 59). Yet making a panel of hybridomas by screening and selecting only high affinity binders may not be enough to distinguish between protective and non-protective anti-Ids (59).

The advantages of polyclonal vs. monoclonal antibodies has recently been reviewed (60). Previous discussions have suggested a soluble antigen reflective of multiple epitopes can be a more potent modulator of humoral and cellular immune responses than Ab2 that represents a singular epitope (61). Counter arguments have been made (62). However, these arguments often neglect a possible influence of a network and the structural basis for antibody recognition. The major characteristic of polyclonal responses is their clonal and structural diversity. Multi-epitope binding increases the overall avidity to the target. For optimizing the targeting of Ab2 to idiotype expressing B-cell receptors all classes of anti-Id, (Ab2α, Ab2β, Ab2γ, and Ab2δ) should be involved. Thus, a polyclonal or oligoclonal anti-Id vaccine would improve targeting, by invoking a “normal' polyclonal immune response. Polyclonal B cell response is a natural mode of an immune response in adaptive immunity. It is a practical and functionally important element of a healthy immune system, with considerable evidence to support its role in protection from at the least infectious agents. Consequently, we are proposing to change the strategy of monoclonal-based anti-Id vaccine development and use. Immunizing with polyclonal–based anti-Ids has the capacity to induce humoral antigen spread in patients by engaging multiple BCR's with the potential to activate both targeted and non-targeted antibody producing B and T cells. Immunizations with selected polyclonal anti-Ids to one or multiple target antigens might be a plausible strategy to amplify preexisting B cells and potentially preexisting T cell responses in addition to *de novo* generation of novel responses. This strategy abandons the concept that the idiotype vaccine represents the “Internal Image” of the antigen and supports our earlier suggestion of being a “Network Antigen” (63).

## 5. Recipes for Making Polyclonal Anti-Id-Based Vaccines

A key prerequisite for an idiotypic network is poly/autoreactivity of some B cell clones. Moreover, it implies positive selection on existing variable regions for which there is evidence (64–66). Positive selection of the B cell repertoire has been demonstrated numerous times over a span of years (67–71) but the nature and the intensity of the self-signal define the choice between elimination, annergy and survival. This implies that a certain range of signal intensities including from existing antibody variable regions can probably recruit the emergent repertoire (7). A constant component of natural IgM would provide the necessary signal exposing idiotopes in the CDR3 regions (72), albeit other regions can be defined as idiotope containing (73), of the required concentration. The unique structures would be too dilute but those shared by a number of clones or sets of clonal products recognized cross-reactively by the same paratope would provide signal sufficient either for positive selection or for negative if the signal were too strong. Maybe this precludes the selection by too broadly distributed public idiotopes. It is interesting to speculate that every strong antibody response might temporarily provide a similar signal. During this time of optimal intensity it may recruit corresponding anti-idiotypic immature B cells. This mechanism may constitute an indirect way to elicit anti-Ids by (inadvertently) manipulating the existing natural antibody network and its capacity to recruit anti-Ids. It may reconcile the “second generation” network concept (7) with experimental induction of anti-Ids as well as introduce the notion that a set of clones rather than a single antibody may be necessary to put this machine in motion.

To stimulate and simulate a polyclonal response, Ab2s can be a mixture of monoclonal antibodies stimulating B and T-cells (53, 58). There are examples of anti-Ids containing both B and T cell epitopes (59, 74). Admixing them might broaden a response. An alternative concept of inducing antibodies against multiple tumor-associated antigens is a pan-immunogen, which harbors “fuzzy” mimicking determinants to induce a polyclonal response to multiple antigens. This concept has not been developed with an Ab2-based vaccine but antigen-mimicking peptides of glycans and TACA have shown such an ability in preclinical (75–80) and clinical studies (81, 82) where a carbohydrate mimetic peptide can induce polyclonal responses to two or more TACAs (81–83). This can be due both to shared epitopes as well as to a multifaceted mimotope exposing diverse antigenic determinants—a structural substrate of immunological polyspecificity.

The advantage of monoclonal antibodies over polyclonal is its consistency and excellent characterization. Monoclonals are produced by cell cultures seeded from a reference cell bank. In contrast polyclonal antibodies are derived from immunized animals producing a unique batch-specific biochemical and biophysical property. For use in humans, each batch must be validated satisfying the advertised criteria. The call for polyclonal or oligoclonal anti-Id antibodies must be answered with novel production strategies. The final step in monoclonal antibody production by hybridoma or recombinant technologies is the selection of the most potent clone or cell line. This is performed under so-called limiting dilution conditions. Suppose one reduced the stringency of selection and mixed a number of clones including ones with lower affinity. The number of antibodies in this polyclonal mix can be controlled. A master cell bank can be established, similar to the master banks in monoclonal production. However, since there is no experience with the clonal stability of cell lines growing in large cell culture tanks research will be required to maintain the original cell culture mix.

## Covered in This Review

1. Rationale and strategy of idiotype-based vaccines-Sections 1-5.
2. Ab1 can also be used to initiate idiotype cascades - Section 2.
3. Lessons learned - Section 3
  - Ab3 can share binding similarities with Ab1
  - Utility in combination therapy
  - Clinically, Anti-Ids can induce B and T-cell immune responses against antigens.
4. Rational for importance of polyclonal responses - Section 4.
5. Redefining the mimetic nature of anti-Ids as network antigens - Section 4.
6. Introduction of a Master Bank for Polyclonal anti-Ids - Section 5.

## Author Contributions

HK originally laid out the framework and draft of this review. AP contributed to the discussion on polyclonal immunology. TK-E provided clinical assessment of previously published work. All authors contributed to the writing of the manuscript.

### Conflict of Interest Statement

TK-E and AP are named as inventors on an institutional patent application filled by UAMS that is related to the CMP vaccine briefly described in this manuscript. Therefore, TK-E and AP and UAMS have a potential financial interest in the vaccine described. No financial or other support of any kind has resulted from this patent application. These financial interests have been reviewed by approved supervision in accordance with the UAMS conflict of interest policies. The remaining author declares that this research was conducted in the absence of any commercial or financial relationships that could be construed as a potential conflict of interest.
